# Tc99m MDP bone SPECT in a case of osteomyelitis of the skull

**DOI:** 10.4103/0972-3919.63597

**Published:** 2010

**Authors:** Ravinder Singh Sethi, BR Mittal, Anish Bhattacharya, Baljinder Singh

**Affiliations:** Department of Nuclear Medicine, Post Graduate Institute of Medical Education and Research, Chandigarh, India

**Keywords:** Bone SPECT, 99mTc-MDP, osteomyelitis skull

## Abstract

A seven-year-old male child presented with swelling at the left temporal region. His skull X-ray was normal. A three-phase bone scan showed increased blood flow, soft tissue activity, and increased tracer concentration in the left temporal region. Single photon emission computed tomography (SPECT) imaging of the skull revealed full thickness involvement of the left temporal bone. Our case report shows that, in osteomyelitis of the skull, SPECT imaging provides significantly more information for identifying the extent and thickness of bone involvement.

## INTRODUCTION

We report a case where SPECT has shown an incremental value over conventional three phase Bone Scintigraphy in diagnosing osteomyelitis of skull

## CASE REPORT

**Figure 1:**
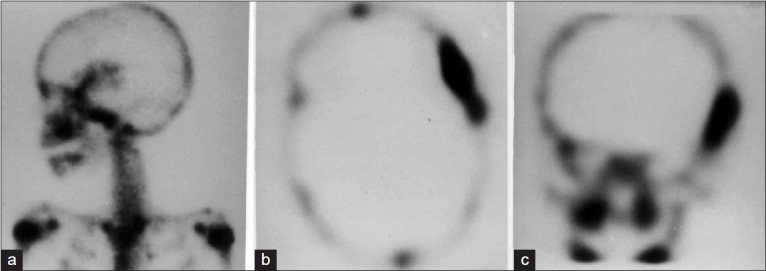
Left lateral view of the skull (a) of the Tc99m MDP static planar bone scan showing an oval area of increased tracer concentration in the left temporal region. Transverse section (b) and Sagittal view (c) of bone the SPECT of the skull showing the extent of the increased tracer concentration in the left temporal region involving full thickness of the bone

A seven-year-old male child presented with a swelling of two months' duration in the left temporal region. It was not associated with fever and there was no history of trauma. On examination, the swelling was 4 × 4 cm in size and hard in consistency. The skull X-ray was normal. Fine needle aspiration cytology (FNAC) was suggestive of inflammation. However, no response was noticed with broad spectrum antibiotics given for two weeks. Subsequently, a three-phase bone scan done with 99mTc-MDP (Methylene Diphosphonate) showed increased blood flow and soft tissue tracer concentration in the left temporal region. Delayed static images also showed increased tracer concentration in the left temporal region [Figure 1a]. However, to determine the full extent of bone involvement, a SPECT study of the skull was performed, which revealed increased tracer concentration in the left temporal region, involving full thickness of the bone [Figures 1b and c]. Finally a diagnosis of acute inflammatory process involving the left temporal region was made. Later curettage of the swelling was done and the subperiosteal pus collection was drained. Pus culture showed growth of *Staphylococcus aureus*. The patient responded to oral cloxacillin.

## DISCUSSION

Acute osteomyelitis may be of hematogenous origin or may be caused by a direct wound or puncture. Hematogenous osteomyelitis is a relatively common problem in children. Early diagnosis and treatment frequently leads to complete recovery. Early diagnosis may be difficult, because conventional and radiological findings are often nonspecific, subtle or simply absent. Skeletal scintigraphy may establish an early diagnosis and thus lead to therapy before much bone destruction occurs. In a three-phase bone scan, initial hyperemia represents increased blood flow due to inflammation, while delayed bone images demonstrate increased radiotracer concentration.[1,2] Photopenic or cold patterns may also be seen in acute hematogenous osteomyelitis.[3,4] Acute hematogenous osteomyelitis most often involves rapidly growing bone and characteristically localizes in the metaphysis of long bones.[5] In children, the most common sites of osteomyelitis are the head of the femur, humerus head, ileum, ischium, talus, and patella.[4] Osteomyelitis of the skull as demonstrated in this case is an uncommon entity. The focus of infection being near the brain, the urgency in making this diagnosis and starting therapy is self-evident. This case shows how a three-phase bone scan along with the SPECT of the skull were helpful in making the diagnosis of osteomyelitis of the skull and in describing the extent of bone involvement. Skull SPECT is a good adjunctive technique to the three-phase bone scan in such cases.
